# First Report of *bla*_IMP–4_ and *bla*_SRT–2_ Coproducing *Serratia marcescens* Clinical Isolate in China

**DOI:** 10.3389/fmicb.2021.743312

**Published:** 2021-10-01

**Authors:** Xiangning Huang, Siquan Shen, Qingyu Shi, Li Ding, Shi Wu, Renru Han, Xun Zhou, Hua Yu, Fupin Hu

**Affiliations:** ^1^Department of Laboratory Medicine, Sichuan Provincial People’s Hospital, University of Electronic Science and Technology of China, Chengdu, China; ^2^Huashan Hospital, Institute of Antibiotics, Fudan University, Shanghai, China; ^3^Key Laboratory of Clinical Pharmacology of Antibiotics, Ministry of Health, Shanghai, China

**Keywords:** *Serratia marcescens*, *bla*_IMP–4_, *bla*_SRT–2_, IncN plasmid, class 1 integron

## Abstract

Carbapenem-resistant *Enterobacterales* (CRE) has become a major therapeutic concern in clinical settings, and carbapenemase genes have been widely reported in various bacteria. In *Serratia marcescens*, class A group carbapenemases including SME and KPC were mostly identified. However, there are few reports of metallo-β-lactamase-producing *S. marcescens.* Here, we isolated a carbapenem-resistant *S. marcescens* (S378) from a patient with asymptomatic urinary tract infection which was then identified as an IMP-4-producing *S. marcescens* at a tertiary hospital in Sichuan Province in southwest of China. The species were identified using MALDI-TOF MS, and carbapenemase-encoding genes were detected using PCR and DNA sequencing. The results of antimicrobial susceptibility testing by broth microdilution method indicated that the isolate *S. marc*escens S378 was resistant to meropenem (MIC = 32 μg/ml) and imipenem (MIC = 64 μg/ml) and intermediate to aztreonam (MIC = 8 μg/ml). The complete genomic sequence of *S. marcescens* was identified using Illumina (Illumina, San Diego, CA, United States) short-read sequencing (150 bp paired-end reads); five resistance genes had been identified, including *bla*_IMP–4_, *bla*_SRT–2_, *aac(6′)-Ic*, *qnrS1*, and *tet(41)*. Conjugation experiments indicated that the *bla*_IMP–4_-carrying plasmid pS378P was conjugative. Complete sequence analysis of the plasmid pS378P bearing *bla*_IMP–4_ revealed that it was a 48,780-bp IncN-type plasmid with an average GC content of 50% and was nearly identical to pP378-IMP (99% nucleotide identity and query coverage).

## Introduction

*S. marcescens* is recognized to be an important nosocomial pathogen and is usually associated with outbreaks in neonatal wards (Mahlen, 2011; Millán-Lou et al., 2021). The infection caused by *S. marcescens* can cause nosocomial infection, affecting several parts of the body, such as the meninges, blood, and lungs, leading to a series of infections like central nervous system infections, blood infections (including endocarditis), and nosocomial pneumonia (Mahlen, 2011; da Silva et al., 2021). The emergence of multidrug-resistant (MDR) *S. marcescens* strains poses a serious threat to public health. One important feature of *S. marcescens* is its intrinsic and acquired resistance to a large number of antibiotics including ampicillin, nitrofurantoin, tetracycline, macrolides, cefuroxime, cephamycin, fluoroquinolone, and colistin (Sandner-Miranda et al., 2018). The identification of carbapenem-resistant *S. marcescens* in patients might pose potential spread into the hospital environment and/or to other patients. For example, in 2020, the outbreak of KPC-3-producing *S. marcescens* among nursing institutions in the United States made it very difficult for clinical treatment (Jimenez et al., 2020). The spread of carbapenem-resistant *S. marcescens* in a hospital environment is a worrying problem.

The metallo-β-lactamases (MBLs) can hydrolyze nearly all β-lactams, and their activities cannot be inhibited by clinically available β-lactamase inhibitors including avibactam, relebactam, and vaborbactam (Wu et al., 2019; Boyd et al., 2020). IMP-type MBLs were the earliest transferable carbapenemases reported in Gram-negative bacteria (Watanabe et al., 1991). Until now, more than 82 variants of *bla*_IMP_ have been identified,^1^ *bla*_IMP–4_, as the most reported IMP variant, has often been found in class 1 integrons and carried by multiple plasmid types like HI2, L/M, A/C, and N for dissemination (Lai et al., 2017; Wang et al., 2018; Roberts et al., 2020). However, unlike NDM-type MBLs, *bla*_IMP_ was not commonly detected among CRE from China (Zhang et al., 2017; Wang et al., 2018; Han et al., 2020). According to a longitudinal large-scale CRE Study in China from 2012 to 2016 (65 hospitals in 25 provinces were included), the common species carrying *bla*_IMP–4_ were *Enterobacter cloacae*, *Escherichia coli*, *Klebsiella pneumoniae*, and *Citrobacter freundii* (Wang et al., 2018). In 2005, the first clinical isolate of *bla*_IMP–4_-positive *S. marcescens* was identified in Australia (Peleg et al., 2005). In this study, we reported a *bla*_IMP–4_ and *bla*_SRT–2_ positive *S. marcescens* containing an IncN-type plasmid in China.

## Materials and Methods

### Strains and Antimicrobial Susceptibility Testing

A carbapenem-resistant *S. marcescens* (S378) was isolated from a urine sample from a patient with asymptomatic urinary tract infection at a tertiary hospital in Sichuan Province in southwest of China. Species identification was performed using MALDI-TOF MS (bioMérieux, France). Phenotypic and genotypic detection of carbapenemases was performed using imipenem-EDTA double-disk synergy test and *NG*-Test Carba 5, respectively. The existence of the carbapenemase genes (KPC, NDM, OXA, IMP, and VIM) was confirmed by PCR-based sequencing, as previously described (Gülmez et al., 2008; Woodford et al., 2008; Feng et al., 2016; Ferreira et al., 2020; Solgi et al., 2020; Nikibakhsh et al., 2021). The broth microdilution method recommended by the Clinical and Laboratory Standards Institute (CLSI) was used as a reference for determining the minimal inhibition concentration with quality control and interpretation of the results according to CLSI M100-31th breakpoints for all agents with the exception of tigecycline, polymyxin, and cefoperazone–sulbactam (Clinical and Laboratory Standards Institute, 2021). Cefepime–zidebactam and cefepime–tazobactam referred to criteria for cefepime in CLSI. Tigecycline MICs were interpreted using US FDA MIC breakpoints for *Enterobacterales* (FDA, 2021), and polymyxin MICs were interpreted using the European Committee for Antimicrobial Susceptibility Testing (EUCAST) MIC breakpoints for *Enterobacterales*. *E. coli* ATCC 25922 and *Pseudomonas aeruginosa* ATCC 27853 were used as controls for testing antimicrobial susceptibility.

### Conjugation and Plasmid Sequencing

A conjugation experiment was performed to explore the transferability of the plasmid. Briefly, the *bla*_IMP–4_-positive isolate *S. marc*escens S378 was used as the donor, while the *E. coli* J53 (azide resistant) was used as the recipient strain. Conjugants were selected on Mueller–Hinton (MH) agar supplemented with azide (100 μg/ml) and ampicillin (50 μg/ml). The presence of the *bla*_IMP–4_ gene and other resistance genes in conjugants was confirmed by antimicrobial susceptibility, PCR, and DNA sequencing. The plasmids of the *bla*_IMP–4_-containing conjugants were extracted using the Qiagen Midi kit (Qiagen, Hilden, Germany) and sequenced using Illumina NovaSeq (Illumina, San Diego, CA, United States) short-read sequencing (150-bp paired-end reads). The sequencing reads were trimmed with sickle (GitHub) and *de novo* assembled using SPAdes 3.12.0. To evaluate and compare the assembly results, Pilon 1.18 is used for basic calibration. Open reading frame prediction and annotation were done with RAST version 2.0^2^ and BLAST^3^ at NCBI; the plasmid replicon was determined using the PCR-based replicon typing method (Carattoli et al., 2005). Plasmid comparisons were performed using BRIG^4^ (Alikhan et al., 2011).

### Whole-Genome Sequencing and Bioinformatics Analysis

Genomic DNA was extracted using a Genomic DNA Isolation Kit (Qiagen, Hilden, Germany) and sequenced using Illumina (Illumina, San Diego, CA, United States) short-read sequencing (150-bp paired-end reads). Sequences were *de novo* assembled using SPAdes 3.12.0. Antimicrobial resistance gene analysis and draft genome annotation were performed using BacWGSTdb.^5^

## Results

### Overview of the *bla*_IMP–4_-Positive Isolates

*S. marcescens* S378 was isolated from a urine specimen of a 76-year-old male patient who was admitted to the hospital for treatment of chronic obstructive pulmonary disease. During hospitalization, the patient was dizzy accompanied by shortness of breath and aggravated after activity. CT showed inflammatory changes in the lungs. The patient suffered from subarachnoid hemorrhage and has been cured. Comorbidities of the elderly patient included diabetes mellitus II, hypertension, acute cerebral infarction, occlusion of right internal carotid artery, chronic obstructive pulmonary disease, and asymptomatic urinary tract infection. The interventional operation was performed for this patient to deal with acute cerebral infarction, and on the third day after the operation, he developed a fever and one extended-spectrum β-lactamase-negative *K. pneumoniae* was isolated from the sputum. With suspicion of pneumonia, the patient was started administering intravenous moxalactam (1 g Q12) for 5 days and ceftazidime (1 g Q8h) for 4 days. Subsequently, the patient’s body temperature returned to normal and the infection was controlled. On the 20th day, *S. marc*escens S378 was isolated from the urine causing asymptomatic urinary tract infection. No antibiotic was given for this strain because this *Serratia marcescens* was considered to only colonize in the urinary tract. The patient was recovered and discharged 26 days after admission.

The antimicrobial susceptibility profiles of *S. marc*escens S378 are presented in Table 1. The isolate was susceptible to tigecycline (MIC = 0.5 μg/ml), amikacin (MIC = 16 μg/ml), and Trimethoprim-sulfamethoxazole (MIC ≤ 0.25 μg/L), intermediate to aztreonam (MIC = 8 μg/ml), but resistant to cefoperazone–sulbactam (MICs > 128 μg/ml), meropenem (MIC = 32 μg/ml), imipenem (MIC = 64 μg/ml), ceftazidime-avibactam (MIC ≥ 32 μg/ml), levofloxacin (MIC = 2 μg/ml), and ciprofloxacin (MIC = 1 μg/ml).

**TABLE 1:** Susceptibility of *S. marcescens* clinical isolate, conjugant and recipient to antimicrobial agents.

Strains	β-Lactamase genes	MIC (mg/l)															
		CZA	IPM	MEM	CAZ	FEP	TZP	CSL	ATM	AMK	FPZ	FPT	SXT	LEV	CIP	TGC	POL
*S. marcescens* S378	*bla*_IMP–4_, *bla*_SRT–2_	>32	64	32	>32	>128	>256	>128	8	16	4	>64	≤0.25	2	1	0.5	>16
*E. coli* S378-C	*bla*_*IMP*–4_	>64	2	2	>32	8	8	64	≤1	≤1	0.125	8	≤0.25	1	1	0.25	0.25
*E. coli* J53	−	0.25	0.25	≤0.03	0.5	≤0.06	4	≤1	≤1	≤1	0.06	≤0.03	≤0.25	0.125	≤0.06	0.125	0.25

*CZA, ceftazidime-avibactam; IPM, Imipenem; MEM, meropenem; CAZ, ceftazidime; FEP, cefepime; TZP, piperacillin-tazobactam; CSL, cefoperazone-sulbactam; ATM, aztreonam; AMK, amikacin; FPZ, cefepime-zidebactam; FPT, cefepime-tazobactam; SXT, trimethoprim-sulfamethoxazole; LEV, levofloxacin; CIP, ciprofloxacin; TGC, tigecycline; POL, polymyxin B.*

### Carbapenemase-Encoding Genes and Conjugation Experiments

PCR-based sequencing demonstrated the presence of *bla*_IMP–4_ in *S. marcescens* strain S378. The *bla*_IMP–4_-carrying plasmid was successfully transferred from *S. marcescens* strain S378 to *E. coli* J53, making the conjugants resistant to ceftazidime and ceftazidime–avibactam but intermediate to imipenem and meropenem. Compared with the recipient *E. coli* J53, the meropenem, imipenem, and ceftazidime–avibactam MICs of conjugants increased at least 60-, 8-, and 256-fold, respectively (Table 1).

### Whole-Genome Sequencing Analysis

**FIGURE 1:**
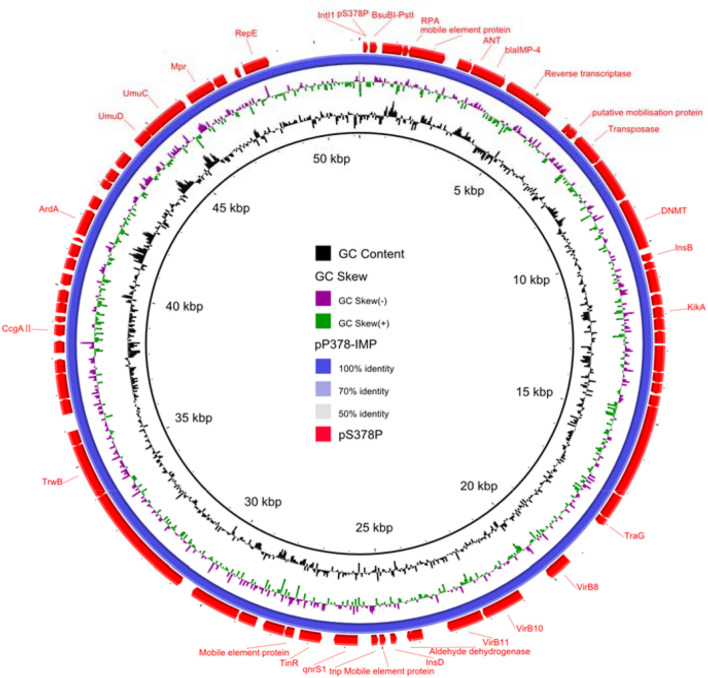
Circular comparison between plasmid pS378P (MZ643942, in this study) and plasmid pP378-IMP (KX711879). The different colors indicated different plasmids and are listed in the color key.

**FIGURE 2:**
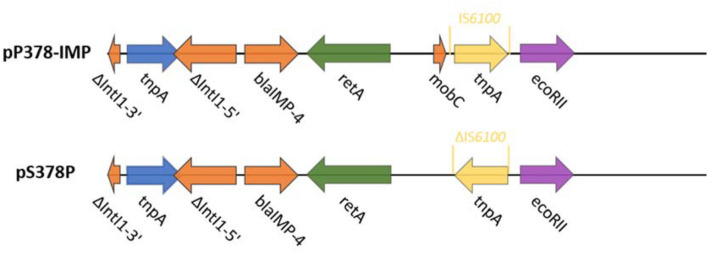
Plasmid accessory resistance regions. The genetic environment comparison of *bla*_IMP–4_ between pP378-IMP and pS378P. Genes are denoted by arrows and colored based on gene function classification.

According to the whole-genome sequencing (WGS) analysis, many resistance genes had been identified, including the β-lactamase genes *bla*_IMP–4_ and *bla*_SRT–2_, the aminoglycoside resistance genes *aac(6′)-Ic*, the fluoroquinolone resistance gene *qnrS1*, and the tetracycline resistance gene *tet(41)*. According to the sequencing results of pS378P, it was a 48,780-bp plasmid (Figure 1), belonging to the IncN type, with an average GC content of 50%. This targeted plasmid contained 43 open reading frames (ORFs). Only two resistance genes were identified in pS378P, *bla*_IMP–4_, and *qnrS1*, conferring resistance to carbapenems and quinolones, respectively. Blast comparison indicates that pS378P in this study shares extensive similarity with pP378-IMP (99% nucleotide identity and query coverage), an IncN-type *bla*_IMP–4_ carrying plasmid with the length of 51,207 bp in a carbapenem-resistant *P. aeruginosa* strain P378 isolated from a teaching hospital in Chongqing China in 2016 (Feng et al., 2016). Like the source of our strain, they were all isolated from urine specimen. pP378-IMP and pS378P both possess the conserved IncN1-type backbone regions, the *tra* genes and *kikA-korB* for conjugal transfer, and IS*1* remnant (Figure 1). There are two major genetic differences between the backbones of pS378P and pP378-IMP. First, pP378-IMP contains an intact anti-restriction gene combination ccgA I, ccgAII, ccgC, and ccgD (located *around* the 4.1–4.6-kb nucleotide positions of pP378-IMP), while only ccgAI and ccgAII genes were found in pS378P. Second, compared with the plasmid pP378-IMP, the class one integron in pS378P is incomplete that an insertion sequence, *IS*6100, was truncated (Figure 2).

## Discussion

According to a previous epidemiological study, the most frequent carbapenemases found in *S. marcescens* species belong to the class A group, including chromosomal location SME type or KPC-2 (Dabos et al., 2019). *bla*_SRT–2_, an AmpC-type β-lactamase gene, was first reported in a *S. marcescens* strain in 2004. Almost all subsequent reports about it are related to *S. marcescens*. Moreover, in *S. marcescens*, *bla*_SRT–2_ often appears with different resistance genes, such as *bla*_CTX–M–3_, *bla*_TEM–1_, aminoglycoside AAC (6′)-Ic, and *bla*_KPC–2_ (Wu et al., 2004; Yu et al., 2008; Srinivasan and Rajamohan, 2019; Quezada-Aguiluz et al., 2020). *bla*_IMP–4_ was first identified in *Acinetobacter* spp. in 2001 from Hong Kong, China (Chu et al., 2001), and had spread rapidly around the world (Espedido et al., 2008; Lee et al., 2017, 2018; Ghaith et al., 2018), but unlike KPC-type and NDM-type carbapenemases among CRE (NDM-type MBL remains predominant), *bla*_IMP–4_ has not been frequently detected, especially in *S. marcescens* (Xiong et al., 2016; Han et al., 2020). In 2018, the first report of *bla*_IMP–4_- and *bla*_VIM–2_-producing *S. marcescens* was published in Egypt (Ghaith et al., 2018). This is a clinical retrospective study. A total of 40 strains of *S. marcescens* were isolated from March to August 2015, of which 42.5% was IMP-4-positive and 37.5% was VIM-2-positive. Just like the strain in our study, they all showed resistance to meropenem and ceftazidime. Our study demonstrates the emergence of carbapenemase-producing *S. marcescens*, expressing *bla*_IMP–4_ and *bla*_SRT–2_ β-lactamase genes in China.

*S. marcescens* is featured by its rapid acquisition of antibiotic resistance, mainly due to the acquisition of plasmid (Mahlen, 2011). However, comprehensive analysis for the genome sequence carrying *bla*_IMP–4_ is rare. According to current reports, although different types of plasmids had been detected, the IncN type remains predominant in China, especially for the transmission of *bla*_IMP–4_ in recent years, and this type of plasmid usually presents a broad host range (Feng et al., 2016; Wang et al., 2017; Xu et al., 2020), compared with the most identical plasmid pP378-IMP. The strains in both studies all harbored a conjugative *bla*_IMP–4_-carrying plasmid, which accounts for the carbapenem resistance phenotype. In both plasmids, the *bla*_IMP–4_ gene was found in class 1 integron, identical to the IMP-4-carrying plasmids before (Xu et al., 2020; Liu et al., 2021). Class 1 integrons are responsible for the transmission of the *bla*_*IMP*_ gene; so far, many Class 1 integrons carrying the *bla*_IMP–4_ gene have been reported, such as In 809, 823, 1,456, 1,460, and 1,589 (Lee et al., 2017; Matsumura et al., 2017; Wang et al., 2017; Dolejska et al., 2018). In addition to integrons and the conjugative plasmids, the insertion sequence also plays an important role in the transmission of resistant genes. Previous studies about *bla*_IMP–4_-carrying plasmids emphasized the role of the IS*26* mobile element, which may play an important role in the dissemination of IMP-4 in different plasmids (Xu et al., 2020; Liu et al., 2021); the corresponding situation also exists in our strains. Timely determination of the resistance mechanism and the transmission mechanism of resistance genes is very important for clinical anti-infective treatment and controlling the wide spread of these multi-drug resistant bacteria.

## Conclusion

In summary, we first identified a *bla*_IMP–4_ and *bla*_SRT–2_ co-positive *S. marcescens* strain from a human urine sample in China. The patient was accompanied by many underlying diseases such as diabetes, emphysema, diabetic peripheral neuropathy, and atherosclerosis, and multiple antimicrobial substances were used in the course of treatment; since such risk factors for MDR bacteria are commonly present in high-risk populations, it seems justified to screen Gram-negative bacilli for carbapenemases in these patients with high-risk factors based on our routine antimicrobial susceptibility testing and molecular biotechnology. Moreover, to date, *S. marcescens* and many other *Enterobacteriaceae* bacteria that are not often reported might still be a neglected source of undetected carbapenemase allocation.

## Data Availability Statement

The datasets presented in this study can be found in online repositories. The names of the repository/repositories and accession number(s) can be found below: GenBank MZ643942.

## Ethics Statement

The study protocol was approved by the Institutional Review Board of Huashan Hospital, Fudan University (No. 2018-408).

## Author Contributions

FH and HY designed the study. SS and XH collected clinical samples and performed the experiments. LD, SW, RH, and XZ analyzed the data. SS and XH wrote the manuscript. All authors contributed to the article and approved the submitted version.

## Conflict of Interest

The authors declare that the research was conducted in the absence of any commercial or financial relationships that could be construed as a potential conflict of interest.

## Publisher’s Note

All claims expressed in this article are solely those of the authors and do not necessarily represent those of their affiliated organizations, or those of the publisher, the editors and the reviewers. Any product that may be evaluated in this article, or claim that may be made by its manufacturer, is not guaranteed or endorsed by the publisher.

## Footnotes

1
http://www.ncbi.nlm.nih.gov/pathogens/beta-lactamase-data-resources/

2
https://rast.nmpdr.org

3
https://blast.ncbi.nlm.nih.gov/Blast.cgi

4
http://brig.sourceforge.net

5
http://bacdb.cn/BacWGSTdb/analysis_single.php
